# Insights into Personalized Care Strategies for Wilms Tumor: A Narrative Literature Review

**DOI:** 10.3390/biomedicines12071455

**Published:** 2024-06-30

**Authors:** Salma Karam, Ahmad Gebreil, Ahmed Alksas, Hossam Magdy Balaha, Ashraf Khalil, Mohammed Ghazal, Sohail Contractor, Ayman El-Baz

**Affiliations:** 1 Bioengineering Department, University of Louisville, Louisville, KY 40292, USA; srkara81@students.campbellsville.edu (S.K.); ahmed.gibreil@louisville.edu (A.G.); ahmed.alksas@louisville.edu (A.A.); hossammagdyhassanhussien.balaha@louisville.edu (H.M.B.); 2 College of Technological Innovation, Zayed University, Abu Dhabi 4783, United Arab Emirates; ashraf.khalil@zu.ac.ae; 3 Electrical, Computer, and Biomedical Engineering Department, Abu Dhabi University, Abu Dhabi 59911, United Arab Emirates; mohammed.ghazal@adu.ac.ae; 4 Department of Radiology, University of Louisville, Louisville, KY 40202, USA; sohail.contractor@louisville.edu

**Keywords:** Wilms tumor, pediatric oncology, personalized medicine, targeted therapy, chemotherapy, radiation therapy, immunotherapy, genetic profiling

## Abstract

Wilms tumor (WT), or nephroblastoma, is the predominant renal malignancy in the pediatric population. This narrative review explores the evolution of personalized care strategies for WT, synthesizing critical developments in molecular diagnostics and treatment approaches to enhance patient-specific outcomes. We surveyed recent literature from the last five years, focusing on high-impact research across major databases such as PubMed, Scopus, and Web of Science. Diagnostic advancements, including liquid biopsies and diffusion-weighted MRI, have improved early detection precision. The prognostic significance of genetic markers, particularly WT1 mutations and miRNA profiles, is discussed. Novel predictive tools integrating genetic and clinical data to anticipate disease trajectory and therapy response are explored. Progressive treatment strategies, particularly immunotherapy and targeted agents such as HIF-2α inhibitors and GD2-targeted immunotherapy, are highlighted for their role in personalized treatment protocols, especially for refractory or recurrent WT. This review underscores the necessity for personalized management supported by genetic insights, with improved survival rates for localized disease exceeding 90%. However, knowledge gaps persist in therapies for high-risk patients and strategies to reduce long-term treatment-related morbidity. In conclusion, this narrative review highlights the need for ongoing research, particularly on the long-term outcomes of emerging therapies and integrating multi-omic data to inform clinical decision-making, paving the way for more individualized treatment pathways.

## 1. Introduction

Wilms tumor (WT) is the most common kidney cancer in children, presenting significant diagnostic and therapeutic challenges. Annually, approximately 500 new cases are diagnosed in the United States [1]. This review synthesizes recent advancements in WT management, focusing on personalized medicine’s transformative potential [2]. With a global incidence influenced by genetic and environmental factors, the review highlights key developments in personalized care. The 5-year survival rate for localized disease exceeds 90% in developed countries [3,4], shifting therapeutic goals towards reducing treatment-related toxicities and addressing outcome disparities related to staging, histology, and socioeconomic factors [5,6,7].

The objectives of this review are:

- To explore WT’s clinical presentations and biological characteristics.
- To evaluate current and emerging therapeutic strategies.
- To illuminate molecular genetics’ role in refining patient management.

We examine how genetic insights, particularly WT1 mutations and miRNA profiles, enhance prognostication and enable patient-specific interventions [8,9]. The review also evaluates novel agents in clinical trials, such as HIF-2α inhibitors and GD2-targeted immunotherapy, for their potential to reshape WT treatment. A critical analysis of current staging practices is provided, advocating for molecular diagnostic enhancements to refine traditional staging systems like the traditional Société Internationale d’Oncologie Pédiatrique (SIOP) and Children’s Oncology Group (COG) staging systems. This review identifies key knowledge gaps and future research areas, emphasizing the need for effective treatments for high-risk patients and strategies to minimize long-term treatment effects. Our intent is to serve as a comprehensive resource for WT management and a catalyst for continued innovation in this evolving field.

Search Strategy/Terms:

We conducted a comprehensive literature search for our narrative review across several major databases, including PubMed, Scopus, and Web of Science. Our search strategy was designed to capture a broad spectrum of high-impact research relevant to WT, particularly focusing on advancements in personalized care. While our review is narrative in nature, we aimed to ensure a thorough and wide-ranging examination of the subject matter. Additionally, we considered grey literature, including conference proceedings and expert opinions, to ensure a comprehensive overview of current and emerging strategies in WT management.

Our search strategy combined key terms and Boolean operators to comprehensively retrieve relevant literature. The primary search terms included “Wilms Tumor”, “nephroblastoma”, “personalized medicine”, “targeted therapy”, “genomic profiling”, and “emerging therapies”. These terms were used in various combinations with Boolean operators such as “AND” and “OR” to expand the search. For example, a typical search string might have been “Wilms Tumor AND personalized medicine OR targeted therapy AND genomic profiling”. This approach allowed us to capture a wide array of articles pertinent to the evolving landscape of WT treatment.

The inclusion criteria were:

- Publication Date: Articles published within the last five years.
- Language: Articles in English.
- Relevance: Studies that specifically address advancements in personalized medicine for WT, including genetic and molecular diagnostics, novel therapeutic strategies, and prognostic tools.
- Study Type: High-impact research articles, reviews, and clinical trial reports.

Exclusion criteria included:

- Non-English Publications: Articles not published in English.
- Irrelevant Focus: Studies that do not specifically pertain to personalized medicine in WT.
- Low Impact: Articles published in low-impact or non-peer-reviewed journals.

Study Selection Procedure:

Initially, we identified a broad pool of articles using the defined search strategy. Titles and abstracts were screened for relevance, and full texts of potentially eligible studies were retrieved and assessed against the inclusion and exclusion criteria. Discrepancies in study selection were resolved through consensus among the authors.

**Figure 1:**
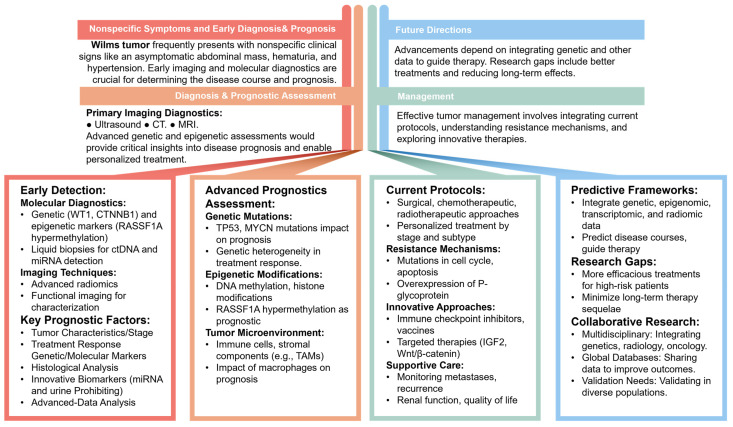
Structure and key concepts of Wilms tumor diagnosis, prognostic assessment, management, and future directions.

The decision to use a narrative review approach stems from the diverse and evolving nature of research in personalized medicine for WT. A narrative review allows for a comprehensive synthesis of recent advancements, integrating findings from various study designs and methodologies. This approach is particularly suitable for capturing the breadth of emerging trends and their clinical implications, providing a holistic overview that can inform future research and clinical practice. A summary of the different key concepts in the field of WT is illustrated in Figure 1.

## 2. Background

WT, or nephroblastoma, while rare, constitutes a significant pediatric nephrology concern, accounting for approximately 7% of all childhood cancers. Epidemiological studies reveal a global incidence of approximately 8–10 cases per million children under age 15, with regional variability suggesting both genetic predisposition and environmental influences at play [5]. Specifically, WT is more prevalent in females and African Americans, highlighting the need to understand underlying socio-genetic mechanisms [10].

The genetic landscape of WT is intricate, with somatic and germline mutations contributing to its etiology. Key genes implicated include WT1, which is associated with syndromic presentations such as WAGR syndrome and CTNNB1, with non-syndromic cases displaying a spectrum of mutations impacting tumor behavior and response to therapy [11]. While less well-defined, environmental factors are an area of ongoing investigation, particularly concerning prenatal exposures [12]. Recent research has revealed that over 40% of relapse samples contain mutations in SIX1 or genes of the MYCN network, drivers of progenitor proliferation. DIS3 and TERT have also been identified as recurrently mutated in relapse samples. Furthermore, mutations in SIX1, MYCN, and WTX are late developments in some individuals, impacting tumor behavior and response to therapy [11].

Clinical manifestations of WT often present a diagnostic challenge due to their heterogeneous nature, with imaging studies such as ultrasound and MRI serving as essential tools in the diagnostic pathway, complemented by histopathological evaluation and emerging molecular diagnostics for precision prognostication [13,14]. The COG Diagnostic Imaging Committee and the SIOP Committee have published a white paper with recommendations for the imaging of pediatric renal tumors [14,15]. Tests and procedures used to diagnose and stage Wilms tumor and other childhood kidney tumors include the following:

- Ultrasound and MRI as essential tools in the diagnostic pathway.
- Emerging molecular diagnostics for precision prognostication.

The therapeutic landscape for WT has undergone a significant evolution, shifting from a one-size-fits-all approach to nuanced, risk-adapted strategies that incorporate histological and molecular features [16]. The utilization of multimodal therapy, including nephron-sparing surgery and tailored chemotherapeutic regimens, has markedly improved survival rates, now exceeding 90% for localized disease [17]. Additionally, exploring targeted therapies, such as HIF-2α inhibitors and GD2-targeted immunotherapy, provides promising avenues for treatment-resistant or metastatic cases, with clinical trials underway to establish their efficacy and safety profiles.

The ultimate objective in treating WT extends beyond achieving high survival rates; it encompasses ensuring the holistic well-being of survivors [6]. Adopting risk-adapted therapeutic strategies marks a significant advancement in the management of WT. Recent statistics reveal that survival rates for localized disease have now exceeded 90% [6]. The most current treatment guidelines from the National Wilms Tumor Study Group (NWTS/COG) and the SIOP mirror this advancement, highlighting the importance of customizing therapy based on the individual patient’s disease characteristics and biology [6]. This evolution in treatment approaches is critical for securing the best possible outcomes for WT patients, aiming to minimize the adverse effects associated with therapy.

## 3. Diagnosis of Wilms Tumor

Clinical Presentation and Diagnostic Challenges

Diagnosing WT can be challenging due to its often subtle and non-specific symptoms, which might include an asymptomatic abdominal mass in young patients. Less frequently, children may exhibit signs such as blood in the urine (hematuria), high blood pressure (hypertension), or abdominal discomfort. These vague symptoms underscore the need for vigilant suspicion and the application of accurate diagnostic tools [18,19].

Imaging and Molecular Diagnostics

The approach to diagnosing WT is comprehensive, incorporating a variety of imaging and molecular diagnostic methods. Ultrasonography (US) is favored as the initial imaging technique because of its non-invasive nature and broad accessibility, offering clear images of the kidney’s structure and any potential tumor-related vascular invasion. For a more detailed analysis of the tumor’s structure, extent, and potential spread to the renal veins, computed tomography (CT) scans are employed. Magnetic resonance imaging (MRI) is also utilized for its superior soft tissue contrast, which is particularly useful in complex cases where the tumor may affect adjacent organs [20,21,22].

Recent advancements in genetic and molecular diagnostics have revolutionized the understanding and characterization of WT. Studies by Hu et al. have identified mutations in the WT1 gene [23], emphasizing its prognostic importance, particularly in bilateral WT cases. Furthermore, the expression of the SIX2 gene alongside DNA methylation patterns has shown diagnostic and prognostic utility [24], offering potential blood-based biomarkers for early WT detection.

Less invasive diagnostic techniques, such as digital droplet polymerase chain reaction (ddPCR) for detecting PIK3CA mutations in body fluids, have shown high sensitivity and the potential for urine and plasma to serve as diagnostic tools for WT. Additionally, liquid biopsies have emerged as non-invasive monitoring tools, detecting somatic mutations in body fluids during diagnosis, with the potential to transform non-invasive monitoring [25].

Innovative approaches in imaging analysis, including semi-automatic segmentation algorithms and 3D reconstruction techniques, have contributed to the volumetric assessment of tumors, preservation of renal parenchyma post-surgery, and enhanced surgical planning, particularly for nephron-sparing surgeries [26].

Moreover, the investigation of long non-coding RNAs like MIAT and the characterization of WT heterogeneity using gene expression profiles and computational algorithms have advanced our understanding of WT at a molecular level, offering potential diagnostic and therapeutic targets [27,28].

Recent advancements in diagnostic technologies are refining our ability to detect Wilms tumor (WT) and laying the groundwork for highly personalized treatment plans. The integration of molecular diagnostics, such as the detection of specific gene mutations and the analysis of gene expression profiles, has begun to inform clinicians about the distinct biological behaviors of WT, enabling a more tailored approach to therapy. For instance, identifying WT1 gene mutations offers critical prognostic information, which can guide decisions regarding the intensity and duration of treatment in bilateral cases. Similarly, non-invasive techniques like liquid biopsies hold the promise of monitoring tumor response to therapy in real time, potentially allowing for dynamic adjustments to treatment regimens. When combined with precise imaging modalities, these molecular insights provide a comprehensive diagnostic profile that supports a stratified treatment approach that balances the efficacy of intervention to minimize long-term adverse effects. As such, the trajectory of diagnostic innovation is not only improving the accuracy of WT diagnosis but is also ushering in an era of personalized medicine where treatment is adapted to the unique genetic and molecular landscape of each patient’s tumor.

Table 1 summarizes more diagnostic advances in WT.

## 4. Histologic Subtypes and Prognostic Implications in Wilms Tumor

### 4.1. Histologic Subtypes of Wilms Tumor

WT, a predominant renal malignancy in children, is characterized by diverse histologic subtypes, each with distinct clinical implications. The most common subtype, favorable histology (FH) Wilms tumor, encompasses approximately 90% of cases and is marked by well-differentiated tissues without anaplastic features. Patients with FH typically have a better prognosis and respond well to standard treatment protocols. In contrast, anaplastic Wilms tumor, identified by the presence of abnormal cell division and large, distorted nuclei, is categorized as unfavorable histology due to its association with a higher risk of treatment failure and recurrence. Other less common subtypes include clear cell sarcoma of the kidney and rhabdoid tumor of the kidney, both of which are associated with poorer outcomes than FH Wilms tumor [35].

### 4.2. Prognostic Implications

The histologic subtype of WT plays a crucial role in determining prognosis, influencing treatment decisions, and predicting outcomes. Favorable histology WT is associated with high survival rates, exceeding 90% with appropriate therapy. However, the presence of anaplastic features significantly alters the treatment approach, often necessitating more aggressive therapy due to its lower survival rates and higher likelihood of recurrence. Clear cell sarcoma and rhabdoid tumor subtypes also demand intensified treatment regimens, reflecting their more aggressive nature and poorer prognosis. Understanding these correlations is vital for tailoring treatment plans to individual patients, aiming to maximize efficacy while minimizing adverse effects, therefore optimizing patient outcomes in this diverse disease [35,36].

## 5. Prognosis and Risk Stratification

Wilms tumor’s prognosis and risk stratification have entered a new era characterized by an intricate blend of traditional clinical indicators and state-of-the-art molecular diagnostics. Recent studies have shed light on essential prognostic indicators, encompassing tumor volume assessment, predictive nomograms, genetic heterogeneity, and the influence of molecular markers on disease recurrence and treatment response. The collaborative efforts of the scientific community are paving the way for personalized medicine in pediatric oncology, ensuring that each patient’s treatment is as unique as their genetic blueprint. Future research will continue to refine these predictive models, aiming to improve survival outcomes and quality of life for children afflicted with Wilms tumor [37].

### 5.1. Tumor Characteristics and Molecular Insights

Advancements in genomic and epigenomic analyses have revolutionized our understanding of Wilms tumor characteristics and their implications for prognosis. Integrating volumetric assessments with molecular markers, such as miRNA profiles and genomic signatures, has led to a more nuanced risk stratification that informs personalized therapeutic approaches.

Emerging molecular insights are increasingly influencing clinical decisions in managing Wilms tumor. For instance, discovering specific gene mutations, such as CTNNB1 and WT1 [38,39], has led to protocols where patients with specific genetic alterations receive targeted therapies, improving their response rates. Molecularly targeted agents, such as inhibitors of the IGF2 and Wnt/β-catenin pathways, are currently being explored in clinical trials to determine their efficacy in WT treatment [40]. These agents have shown promise in preclinical studies and are now being integrated into treatment protocols to enhance therapeutic outcomes. Additionally, epigenomic profiles are being used to identify patients who may have a better prognosis and could be spared from the toxicities of high-dose chemotherapy. Such molecular characterization enables clinicians to make informed, precise, and potentially life-altering treatment choices.

Volume as a Prognostic Indicator and Molecular Correlations: Tumor volume remains a classical prognostic indicator; however, its correlation with molecular characteristics such as specific gene mutations and miRNA dysregulation offers a more sophisticated predictive model [41,42].

#### 5.1.1. Predictive Prognostication and Personalized Therapies

Developing nomograms and bioinformatics tools harnesses large-scale data analysis to deliver precise prognostication and tailored treatment plans. Clinical trials increasingly incorporate these tools to validate their effectiveness in predicting event-free survival and treatment response.

Nomograms and Large-Scale Data: By combining clinical variables with genetic heterogeneity, nomograms facilitate individualized prognosis predictions, while large-scale data analysis continues to uncover novel biomarkers [43,44].

Here is a Proposed Predictive Framework for Individualized Therapy:

#### 5.1.2. Prognostic Markers and Treatment Response

Molecular markers and histology response are central to refining treatment protocols. Identifying biomarkers predictive of recurrence and response to chemotherapy guides the adjustment of treatment intensity and the use of neoadjuvant chemotherapy.

Chemotherapy and Histological Response: Molecular signatures, such as the loss of heterozygosity and gene expression profiles, are critical in predicting the response to chemotherapy and guiding histology-adapted treatment approaches [6,45].

#### 5.1.3. Global Efforts in Advancing Personalized Diagnostics

A global perspective is essential in understanding the variations in tumor biology across diverse populations. International collaborations are key in establishing biology-driven approaches that account for racial and ethnic differences in tumor genetics.

Personalized Care Advocacy and International Collaboration: Through global efforts, researchers advocate for personalized care incorporating biological insights from diverse populations, leading to more equitable and effective treatment strategies.

Predictive tools are invaluable in personalizing treatment strategies for Wilms tumor patients. For example, the use of nomograms incorporating both clinical and genetic data can guide the decision to pursue aggressive treatment in cases with a high risk of recurrence. In contrast, patients with a favorable molecular profile may benefit from more conservative approaches, reducing the risk of overtreatment and its associated morbidities. These examples highlight how sophisticated risk assessment tools directly inform personalized care.

For more research studies focused on prognostic factors in WT, refer to Table 2.

## 6. Prediction

The Horizon of Genomic Prediction

Recent strides in genomic prediction have illuminated the path toward precision medicine in Wilms tumor (WT). Advancements in next-generation sequencing (NGS) and whole-genome sequencing (WGS) have enabled the identification of critical genetic mutations and alterations associated with WT, such as mutations in the WT1, CTNNB1, and WTX genes. These genetic insights have facilitated the development of predictive models that can stratify patients based on their risk profiles, identifying novel predisposition genes beyond the classical WT syndromes., and allowing for more tailored therapeutic interventions [55]. These advancements facilitate the development of genetic panels that can be utilized for risk assessment in asymptomatic individuals, potentially transforming the landscape of early detection and intervention [56].

Syndromic Screening and Beyond

While traditional genetic screening for syndromes associated with WT remains a cornerstone, the focus has shifted toward discovering non-syndromic genetic markers. Syndromic screening is crucial for early detection of Wilms tumor, particularly in patients with genetic syndromes such as Beckwith–Wiedemann syndrome and Denys–Drash syndrome, which are associated with a higher risk of developing the tumor. Early identification through genetic screening and regular surveillance can lead to timely interventions and improved outcomes. Beyond syndromic screening, whole-exome sequencing (WES) and whole-genome sequencing (WGS) are being explored to identify novel genetic markers that could further refine risk stratification and early detection efforts [57].

Imaging and Machine Learning: An Integrative Approach

The fusion of radiomics and machine learning is forging a new frontier in the non-invasive assessment of WT by enhancing tumor detection and characterization accuracy and efficiency. Advanced imaging techniques, such as diffusion-weighted MRI and radiomics, combined with machine-learning algorithms, can analyze complex imaging data to predict tumor histology, treatment response, and prognosis. For example, radiomic features extracted from MRI and CT scans can be used to develop predictive models that distinguish between different tumor subtypes and identify high-risk patients who may benefit from more aggressive treatment. Studies have shown that radiomics combined with appropriate mathematical algorithms have advantages in tumor histological subtype classification, staging, distinguishing lymph node metastases, and prognosis analysis to provide clinical decision support [58,59]. Advanced algorithms that analyze complex imaging data sets distinguish benign from malignant renal masses and predict tumor aggressiveness, stage, and the likelihood of recurrence [59].

MRI-Guided Personalization: Integrating MRI characteristics into predictive models enhances the specificity of treatment regimens. By correlating radiomic features with molecular data, these models can indicate the optimal timing for surgical intervention and the intensity of adjuvant therapy. MRI can accurately delineate tumor boundaries, assess tumor response to preoperative chemotherapy, and detect residual disease, therefore facilitating personalized surgical and radiotherapy strategies. Additionally, MRI-based volumetric assessments can be integrated with molecular data to refine risk stratification and tailor treatment plans [6,60].

Molecular Signatures as Prognostic Beacons

Molecular signatures, encompassing long non-coding RNAs (lncRNAs) and messenger RNAs (mRNAs), have emerged as robust indicators of prognosis. For example, miR-1180-5p regulates apoptosis by targeting p73, and miRNA-19b impacts proliferation and migration through the PTEN/PI3K/AKT pathway. Additionally, circulating serum miRNAs show promise as diagnostic and prognostic biomarkers for WT. These findings underscore the potential of miRNA profiling in improving WT diagnosis and treatment outcomes despite the retraction of specific studies on miR-140-5p [61,62,63]. These signatures, derived from high-throughput genomic analyses, are refining our understanding of tumor biology and patient heterogeneity [64].

Beyond Single Markers: The shift from single-gene analyses to multi-gene signatures and even whole-genome profiles represents a paradigm shift in prognostication. Integrative approaches that combine genomic, transcriptomic, and epigenomic data can identify complex molecular signatures that predict treatment response and disease progression [65]. This comprehensive profiling can inform the development of multi-targeted therapies and personalized treatment plans that address the unique molecular landscape of each patient’s tumor [66]. Through comprehensive molecular profiling, we are moving toward a holistic view of the tumor, enabling a more nuanced prediction of clinical outcomes.

Innovations in Risk Assessment and Prognosis

In an era where personalized medicine is the goal, integrating genomic and imaging data into predictive models revolutionizes prognostic accuracy. Novel predictive models are being developed and validated against clinical outcomes, setting new standards for therapeutic decision-making [67]. For example, circulating tumor DNA (ctDNA) analysis, also known as liquid biopsy, can detect minimal residual disease and monitor treatment response in real time, providing a non-invasive method for risk assessment and early detection of relapse. Machine-learning models integrating clinical, molecular, and imaging data are also being developed to predict patient outcomes and guide personalized treatment strategies [68].

A Framework for the Future: Building on these innovations, we propose a predictive framework encompassing genetic, epigenomic, transcriptomic, and radiomic data. This framework would predict disease courses and guide individualized therapy by identifying patients who may benefit from emerging targeted treatments.

The predictive landscape of Wilms tumor is rapidly evolving, driven by an ever-deepening understanding of tumor genetics and the innovative application of technology. As we stand on the precipice of a new era in pediatric oncology, integrating multi-dimensional data heralds a future where each child’s treatment is tailored to their unique genetic and biological profile, delivering on the promise of truly personalized care [69].

## 7. Management Strategies and Innovations in Wilms Tumor Therapy

Introduction to Modern Management

The treatment landscape of WT has witnessed significant advancements, with innovations that synergize classical methods with precision medicine. Current protocols are intricately designed around tumor stage, histological subtype, and patient-specific factors, reflecting a move toward a highly individualized treatment approach. This section outlines the contemporary management strategies for WT, encompassing the integration of surgical, chemotherapeutic, and radiotherapeutic standards with cutting-edge therapies while emphasizing the importance of holistic, supportive care [2].

Standard Treatment Protocols and Personalization

Standard treatment protocols are personalized based on molecular insights identifying specific genetic and epigenetic alterations in each patient’s tumor. For example, patients with favorable histology and low-risk genetic profiles may receive less intensive chemotherapy, reducing the risk of treatment-related toxicities. Conversely, patients with high-risk genetic alterations, such as WT1 mutations or 1q gain, may benefit from more aggressive treatment regimens and targeted therapies [6,57].

Surgical Techniques with Precision Timing

Surgical resection remains the foundation of WT treatment. Preoperative chemotherapy responses, guided by molecular risk stratification, determine the timing and extent of nephrectomy. SIOP and COG have refined their guidelines to minimize the importance of surgery and preserve renal function whenever possible [70]. Nephron-sparing surgery, which aims to protect kidney function while achieving complete tumor resection, is increasingly being used in selected patients with bilateral or multifocal tumors. Additionally, using intraoperative imaging and navigation systems can enhance surgical precision and reduce the risk of complications [71].

Refined Chemotherapy Regimens

We now understand that the effectiveness of chemotherapy in WT is contingent not just on the stage and histology but also on the genetic and molecular profile of the tumor. Vincristine and actinomycin D remain the mainstay for low-stage tumors, with the addition of doxorubicin for high-risk cases. For example, patients with low-risk tumors may receive reduced-dose chemotherapy, while those with high-risk tumors may receive intensified regimens or novel agents. The incorporation of molecularly targeted agents, such as IGF-1R/AKT signaling pathway inhibitors, is also being explored to enhance treatment response and overcome resistance [72]. Precision medicine has paved the way for dosing adjustments and the introduction of novel agents targeting specific molecular aberrations, reducing long-term toxicity [73].

Tailored Radiotherapy

Advancements in radiotherapy techniques, such as intensity-modulated radiation therapy (IMRT), have allowed for more precise targeting of tumor sites, reducing collateral damage to healthy tissues. This is critical in pediatric patients, where reducing late effects is a primary concern [74].

Pioneering Therapeutic Modalities

New therapeutic modalities are being pioneered to improve treatment outcomes and reduce toxicity. These include molecularly targeted agents, immunotherapies, and genetic and epigenetic interventions. For example, anti-GD2 antibodies and immune checkpoint inhibitors are being investigated in clinical trials for their potential to enhance anti-tumor immune responses in Wilms tumor patients [66].

Molecularly Targeted Agents

Emerging molecularly targeted therapies are exploring pathways implicated in WT pathogenesis, such as IGF2 and Wnt/β-catenin. Clinical trials are underway to determine these agents’ efficacy and their role within existing treatment protocols [75]. Also, agents targeting the IGF-1R/AKT and TGFBRI/SMAD2/3 signaling pathways have demonstrated anti-tumor activity in preclinical studies and are being evaluated in clinical trials for their efficacy and safety in Wilms tumor patients [63].

Advancements in Liquid Biopsy

Emerging liquid biopsy technologies, such as digital droplet polymerase chain reaction (ddPCR) [76,77], circulating tumor DNA (ctDNA) analysis [78], and the detection of circulating tumor cells (CTCs) [78], are at the forefront of transforming the landscape of Wilms tumor diagnostics and management. These approaches offer a non-invasive means to detect tumor-specific genetic and epigenetic alterations, providing insights into tumor biology, prognosis, early detection of relapse, monitoring of minimal residual disease, and identification of actionable mutations, as well as the monitoring of treatment efficacy, therefore guiding personalized treatment strategies [77]. Liquid biopsies hold the promise of early detection, real-time monitoring of disease progression, and the identification of resistance mechanisms to current therapies. However, validation studies of these technologies in Wilms tumor have presented mixed results. While some studies highlight the potential of liquid biopsies for high sensitivity and specificity in detecting tumor-derived genetic material, others point to challenges such as false positives/negatives and the variability in detection rates [77,79]. The critical appraisal of these studies underscores the necessity for more extensive, multicentric trials to establish standardized protocols and confirm the clinical utility of liquid biopsy approaches in the context of WT [80].

Furthermore, while our review touches upon the promising horizon of liquid biopsy technologies in Wilms tumor, it also brings to light the current gaps in comprehensive validation studies. This limitation reflects the nascent stage of these technologies and reinforces the call for more focused research to establish their clinical relevance and reliability in the management of WT [78].

Immunotherapeutic Innovations

Immunotherapy represents a new frontier in WT management, with ongoing research into immune checkpoint inhibitors and vaccines designed to target tumor-specific antigens. Early-phase trials examine the potential to harness the patient’s immune response for more effective tumor control.

Immunotherapy, mainly through WT1-targeting cancer vaccines, has emerged as a promising advancement in managing Wilms tumor (WT). Phase I/II trials have shown that WT1 peptide vaccines are safe and effective, and their combination with HLA class I and II peptides has led to improved clinical outcomes. These vaccines elicit a targeted immune response against WT1-expressing cancer cells, potentially minimizing the risk of relapse and enhancing survival rates, with some patients achieving long-term remission. However, developing these vaccines faces challenges, notably the need for large-scale Phase III trials to confirm their efficacy and safety in the broader patient population. Moreover, the integration of immune checkpoint inhibitors with WT1 peptide vaccines is being explored to potentiate the immune response [81]. Recent advancements in CAR T-cell therapy and immune checkpoint inhibitors have shown potential in treating pediatric solid tumors, including WT [82,83]. Clinical trials are underway to evaluate the safety and efficacy of these immunotherapies in WT patients, with early results indicating promising therapeutic benefits.

As our understanding of tumor immunology deepens, the prospect of combining immunotherapy with personalized treatment plans tailored to the genetic and immunological profiles of individual patients could transform the standard of care for those afflicted with Wilms tumor.

Genetic and Epigenetic Interventions

The therapeutic landscape is expanding to include treatments that correct or modulate genetic and epigenetic changes fundamental to WT development. RNA interference technologies and drugs targeting aberrant DNA methylation patterns are under investigation, intending to reverse the oncogenic processes at the molecular level [79]. Techniques such as CRISPR/Cas9 gene editing and epigenetic modulators are being explored for their potential to correct genetic mutations and alter epigenetic modifications, therefore inhibiting tumor growth and progression. These innovative approaches hold promise for developing more effective and less toxic treatments for Wilms tumor patients [79].

Comprehensive Supportive Care

The paradigm of WT management extends beyond the tumor to address the comprehensive needs of the patient and their family. Ensuring comprehensive care involves diligent follow-up to address any long-term side effects, vigilantly checking for any signs of cancer returning, and safeguarding the health of the kidneys and heart. Providing psychological support is crucial for helping patients and their families navigate the emotional challenges of the cancer experience and adapt to life after treatment [57].

WT treatment is a prime example of how precision medicine is applied in oncology, blending established and innovative treatments to match each tumor’s distinct genetic and biological makeup. As treatment methods continue to advance, there is a strong potential for increasing treatment effectiveness, minimizing harmful side effects, and significantly improving life outcomes for children with cancer, therefore establishing a high standard of care in oncology [6].

Proposed Predictive Framework for Individualized Therapy

To implement a predictive framework encompassing genetic, epigenomic, transcriptomic, and radiomic data, we propose the following steps:

1. Genetic Profiling: Utilize next-generation sequencing (NGS) and whole-genome sequencing (WGS) to identify critical genetic mutations and alterations associated with Wilms tumor (WT), such as WT1, CTNNB1, and WTX mutations. This genetic information can stratify patients based on risk profiles and guide targeted therapeutic interventions.
2. Epigenomic Analysis: Conduct epigenomic profiling to identify DNA methylation patterns and histone modifications that may influence gene expression in WT. This can help understand tumor behavior and potential resistance mechanisms.
3. Transcriptomic Data Integration: Use RNA sequencing (RNA-seq) to analyze gene expression profiles. Differentially expressed genes can serve as biomarkers for prognosis and treatment response, aiding in the customization of therapy.
4. Radiomic Data Utilization: Incorporate advanced imaging techniques such as diffusion-weighted MRI and radiomic analysis to assess tumor characteristics non-invasively. Radiomic features can be correlated with genetic and transcriptomic data to enhance predictive accuracy.
5. Data Integration and Machine Learning: Develop models integrating genetic, epigenomic, transcriptomic, and radiomic data. These models can predict patient outcomes, monitor treatment response, and guide personalized treatment strategies.
6. Clinical Implementation: Establish multidisciplinary teams to interpret integrated data and make informed clinical decisions. Regularly update predictive models with new data from clinical trials and patient registries to refine their accuracy and applicability.

By following these steps, clinicians can implement a comprehensive predictive framework that enhances individualized therapy for WT patients.

## 8. Discussion

In this review, we delve into the significant evolution in the management of WT, focusing mainly on the shift toward a more personalized approach to treatment. This critical change is rooted in the combined use of genetic and phenotypic information, which is increasingly acknowledged as crucial for enhancing outcomes in children with cancer.

Integrating Genetic Information into Clinical Decisions

Breakthroughs in genetic sequencing have been pivotal for identifying genetic alterations linked to WT, impacting both diagnosis and therapeutic strategies. Key mutations in genes like WT1, CTNNB1, and WNT, for example, have been linked to the onset and progression of the tumor, as well as its response to treatment, thus providing new avenues for targeted therapy. Clinical trials progressively leverage genetic insights to categorize patients and customize treatment plans more effectively. Preliminary results from these studies indicate that patients who receive treatment based on their genetic profile experience better event-free survival rates than those treated with conventional methods [74].

Advantages:

- Precision: Allows for more targeted therapies based on individual genetic profiles, improving treatment efficacy.
- Customization: Enables customization of treatment plans, potentially leading to better patient outcomes.

Disadvantages:

- Accessibility: Genetic testing may not be widely available in all clinical settings.
- Cost: High costs associated with advanced genetic testing and targeted therapies.

Refinement of Risk Assessment

Predictive models have evolved to include molecular biomarkers that offer a more refined risk stratification, impacting treatment intensity and modality. For example, specific miRNA signatures have been correlated with treatment resistance, prompting more aggressive chemotherapy regimens in these patients [64]. These approaches are beginning to translate into practice, promising improvements in recurrence rates and long-term survival [63].

Advantages:

- Risk Stratification: Provides a more precise assessment of patient risk, allowing for tailored treatment intensities.
- Improved Outcomes: Correlating molecular biomarkers with treatment resistance can lead to better management of high-risk patients.

Disadvantages:

- Validation: Requires further validation in diverse populations to ensure accuracy and applicability.
- Implementation: Challenges in integrating these models into routine clinical practice due to complexity.

Advancements in Treatment Approaches

Integrating targeted molecular therapies offers a compelling example of personalized medicine’s potential. Agents targeting the IGF2 and Wnt/β-catenin pathways are undergoing clinical evaluation, with preliminary results indicating efficacy in WT subtypes harboring relevant mutations [84]. Such targeted approaches improve survival and aim to minimize the long-term adverse effects associated with conventional chemotherapy [56].

Advantages:

- Efficacy: Potentially higher efficacy in treatment-resistant cases.
- Reduced Toxicity: Lower risk of long-term side effects compared to conventional chemotherapy.

Disadvantages:

- Limited Data: Insufficient long-term data on the safety and effectiveness of these therapies.
- High Costs: Advanced therapies can be expensive and may not be accessible to all patients.

The Role of Imaging and Radiomics

Advanced imaging techniques and the application of radiomics provide non-invasive methods for tumor characterization, which can be coupled with molecular data to guide treatment decisions. Integrative models combining imaging features with genetic information are promising in predicting disease progression and therapeutic response, leading to more personalized surgical and radiotherapeutic planning [71].

Advantages:

- Non-invasive: Provides a non-invasive method for tracking tumor characteristics and treatment response.
- Precision: Enhances the accuracy of staging and treatment planning.

Disadvantages:

- Cost: High cost of advanced imaging techniques.
- Validation: Requires further validation and standardization for routine clinical use.

Clinical Implications and Future Directions

The ultimate goal of personalized medicine in WT management is to ensure that each child receives the most effective and least toxic treatment regimen. Case studies and clinical trials are beginning to illustrate the real-world benefits of this approach. One notable example is the reduction in nephrectomy extent and consequent preservation of renal function in patients with favorable molecular profiles, leading to a better quality of life post-treatment [6].

As we continue to refine these personalized strategies, it is critical that we also focus on the development of international registries and databases, which will allow for the aggregation of data and the validation of predictive models across diverse patient populations. Such collaborative efforts are essential to accelerate the discovery of effective treatments and to democratize access to personalized care for all children with WT, regardless of geographical location.

Personalized medicine in WT represents a confluence of genetic, molecular, and imaging data, driving a more nuanced approach to patient management. While challenges remain in the widespread implementation of these strategies, the evidence to date underscores their potential to revolutionize pediatric oncology. Future research should continue to elucidate the mechanisms underlying tumor heterogeneity, refine predictive models, and validate targeted therapies, all with the aim of individualizing treatment to the genetic and biological landscape of each child’s tumor.

The integration of genomic and radiomic data, as explored by Yu et al. [85], presents a promising avenue for advancing personalized medicine in Wilms tumor. This approach aligns with our review’s focus by potentially enhancing the precision of diagnosis, prognosis, and treatment customization, embodying the future direction of WT management.

## 9. Limitations, Knowledge Gaps, and Future Directions

Despite the advancements in personalized medicine for Wilms tumor, several limitations and knowledge gaps need to be addressed. Some of them are inherent in the individual studies surveyed. These include methodological constraints, such as the variability in study design and data collection methods, which may affect the comparability and reproducibility of findings. Additionally, the representativeness of study populations often varies, with many studies focusing on specific demographic or geographic groups, potentially limiting the generalizability of results to broader patient populations. Furthermore, the scope of research in some studies is narrowly focused on particular aspects of Wilms tumor, which may overlook the multifaceted nature of the disease and its treatment. These limitations underscore the need for cautious interpretation of the findings and highlight the importance of conducting further research that addresses them. These limitations also point towards specific areas where future research is critically needed to improve personalized care in WT, such as:

Genetic and Molecular Heterogeneity: Our current understanding of Wilms tumor’s genetic and molecular landscape is not comprehensive, especially concerning the predictability of treatment response and the impact of ethnic and genetic diversity on disease progression and outcomes [86,87]. Furthermore, while our review touches upon the promising horizon of liquid biopsy technologies in Wilms tumor, it also brings to light the current gaps in comprehensive validation studies. This limitation reflects the nascent stage of these technologies and reinforces the call for more focused research to establish their clinical relevance and reliability in the management of Wilms tumor.

Biomarker Validation: While several potential biomarkers have been identified, large-scale validation studies have not confirmed their clinical utility in risk stratification and treatment personalization [88,89].

Limitations and Validation of Predictive Models:

While nomograms and predictive models offer significant potential in personalizing treatment strategies for Wilms tumor, several limitations must be acknowledged:

1. Population Diversity: Many predictive models are developed using data from specific demographic or geographic populations, which may not represent the broader patient population. This can limit the generalizability of the models.
2. Data Quality and Consistency: The accuracy of predictive models depends on the quality and consistency of the data used. Variability in data collection methods and clinical practices can affect model performance and reliability.
3. Validation in Diverse Populations: Predictive models must be validated in diverse populations to ensure their applicability across racial and ethnic groups. This requires international collaboration and the establishment of global patient registries.
4. Model Complexity and Interpretability: While powerful, complex machine-learning models can be challenging to interpret and implement in clinical practice. To facilitate clinical adoption, efforts should be made to balance model complexity with interpretability.

Treatment Resistance: Understanding the mechanisms underlying treatment resistance in Wilms tumor is crucial for developing effective therapeutic strategies. Several factors contribute to treatment resistance, including:

1. Genetic Mutations: Mutations in genes such as TP53 and MYCN have been associated with resistance to chemotherapy and poor prognosis in WT patients. These mutations can lead to cell cycle regulation and apoptosis alterations, making tumor cells less responsive to treatment.
2. Epigenetic Modifications: Epigenetic changes, such as DNA methylation and histone modifications, can influence gene expression and contribute to treatment resistance. For example, hypermethylation of the RASSF1A gene has been linked to resistance to chemotherapeutic agents in WT.
3. Tumor Microenvironment: The tumor microenvironment, including immune cells, stromal cells, and extracellular matrix components, can impact treatment response. For instance, tumor-associated macrophages (TAMs) have been correlated with resistance to chemotherapy and radiotherapy.
4. Drug Efflux Mechanisms: Overexpression of drug efflux transporters, such as P-glycoprotein, can decrease intracellular concentrations of chemotherapeutic agents, resulting in treatment resistance. Targeting these transporters may enhance the efficacy of chemotherapy.

More research is needed to understand treatment resistance mechanisms, particularly in relapsed or refractory Wilms tumor cases, to develop more effective second-line therapies [90].

Adult-Specific Research: Most of the existing research and subsequent treatment protocols are based on pediatric cases, with limited studies focusing on adult Wilms tumor, which could present differently and may require distinct therapeutic approaches [91].

Global Accessibility: There is a disparity in the accessibility of genomic technologies and personalized medicine between high-income and low—to middle-income countries, impacting the ability to implement advanced treatments universally [92,93].

## 10. Conclusions

In conclusion, the evolution of personalized medicine in the treatment of Wilms tumor (WT) represents a significant milestone in pediatric oncology. The integration of genomic profiling, targeted therapies, and immunotherapy has paved the way for treatments that are increasingly tailored to the unique genetic and molecular characteristics of each patient’s tumor. These advancements hold great promise for improving patient outcomes by enhancing the specificity and efficacy of treatments while minimizing long-term toxicities associated with conventional therapies.

The journey toward truly personalized care, however, is fraught with complexities. Tumor heterogeneity poses a significant challenge, as the genetic and molecular landscape of WT can vary widely among patients. This variability necessitates comprehensive molecular characterization across diverse populations to fully understand the implications of different genetic alterations and to refine risk stratification models. Additionally, disparities in healthcare access, particularly regarding advanced genomic technologies and innovative therapies, must be addressed to ensure that all patients benefit from these advancements, regardless of their socioeconomic or geographic background.

The necessity for a concerted, multidisciplinary approach is underscored by the collective insights from recent studies. Future research must prioritize the validation of potential biomarkers for clinical use, enabling early detection and monitoring of WT and guiding personalized treatment strategies. The exploration of innovative therapies, such as CAR T-cell treatment, should be accelerated, with a focus on overcoming the unique challenges presented by solid tumors.

Equitable access to personalized care remains a critical issue. Reducing socioeconomic disparities that affect access to genomic technologies and advanced therapies is essential. International collaboration is also vital for the aggregation of data, validation of predictive models, and the democratization of personalized care. By fostering such collaboration among clinicians, researchers, policymakers, and patient advocacy groups, we can ensure that the benefits of personalized medicine are extended to all children with WT, regardless of their geographic location.

As we stand at the crossroads of potential and uncertainty, the progress made thus far provides a solid foundation for future advancements. By continuing to bridge the gaps in our knowledge and capabilities, we can look forward to a future where personalized medicine is not merely an ideal but a reality for patients with Wilms tumor. This will ensure better outcomes, improved quality of life, and renewed hope for those at the heart of our endeavors—our patients. Through collaborative efforts and sustained research, we can realize the promise of personalized medicine, transforming the landscape of pediatric oncology and providing a beacon of hope for the future.

## Figures and Tables

**Table 1:** Summary of more high-impact studies on Wilms tumor (WT), outlining their objectives, methodologies, sample characteristics, key findings, and the implications for advancing personalized care in diagnosing Wilms tumor.

Study	Objective	Methods	Sample	Key Findings	Implications
Hu et al. [29]	Explore WT1 gene mutations in bilateral WT	High-res melting, sequencing, Western blot	8 bilateral WT patients	High rate of WT1 mutations in exon 8; early onset of bilateral WT	Personalized genetic screening could enable earlier and more targeted interventions for patients with a familial predisposition to bilateral WT.
Song et al. [30]	Evaluate the SIX2 gene in WT diagnosis/prognosis	MSP, qRT-PCR, ROC analysis	Tissues from 38 WT patients	AUCs indicate the high diagnostic utility of SIX2 expression in blood	Utilizing blood-based biomarkers such as SIX2 expression can enhance non-invasive screening and early diagnosis, tailoring individual patient management.
Waberski et al. [25]	Assess PIK3CA mutations in PROS and WT	ddPCR on plasma, urine cfDNA	Samples from PROS patients	The high sensitivity of ddPCR for PIK3CA variants; potential urine cfDNA as WT marker	Detecting PIK3CA mutations in urine cfDNA offers a less invasive method for identifying WT, facilitating a personalized approach to diagnosis.
Brillantino et al. [31]	Detect somatic mutations in WT at diagnosis	WES, Ion Reporter variant detection	WT tissues from surgery	Somatic mutations were identified in all cases; urine/plasma was the less invasive monitoring tool	Identifying somatic mutations through less invasive samples like urine or blood enables ongoing, non-invasive monitoring of disease status, which can be personalized to the mutation profile of a patient’s tumor.
Rickard et al. [32]	Semi-automatic volumetric assessment of WT and renal parenchyma	MATLAB-3D volumetric imaging, segmentation	98 CT scans of WT patients	Effective capture of treatment responses; preservation of parenchyma post-surgery	Volumetric imaging allows for personalized and precise assessment of tumor response and kidney preservation, informing tailored surgical strategies.
Chaussy et al. [33]	Utilize 3D reconstruction for WT surgical planning	3D Slicer software, inter-individual Dice index assessment	14 scans from 12 patients	High Dice index for WT and healthy kidney; feasibility of 3D reconstruction for NSS	3D reconstruction for surgical planning can lead to more personalized surgeries that minimize the impact on healthy tissue and improve postoperative outcomes.
Trink et al. [34]	Characterize WT heterogeneity using gene expression	Microarray data, PCA, CPM algorithm	53 CEL files from the GEO database	Identification of distinct WT types and unique cell populations	Characterizing WT heterogeneity through gene expression allows for more precise tumor classification, which can guide tailored treatment decisions.

**Table 2:** An aggregated outline of select research studies focused on prognostic factors in Wilms tumor (WT), detailing their investigative goals, methods utilized, populations studied, fundamental discoveries, and how these findings underscore the imperative to integrate prognostic biomarkers and genetic features into the personalization of diagnosis, prognosis, and treatment planning for WT.

Study	Objective	Methods	Sample	Key Findings	Implications
Provenzi et al. [42]	Examine tumor volume changes post-chemotherapy	Statistical analyses: Chi-square, Kaplan–Meier, Cox regression	Patients from 1989 to 2009	The significant relationship between post-chemotherapy tumor volume >500 mL and survival	Large tumor volume post-chemotherapy reveals a need for potentially more aggressive or alternative treatment paths in personalized care planning.
Tang et al. [43]	Develop nomograms for survival prediction	Cox regression, ROC curves	1613 WT patients (SEER database)	Nomograms predicted 3- and 5-year OS and CSS with AUCs 0.65–0.74	Nomograms aid in creating personalized survival predictions, directly influencing patient-specific treatment decisions.
Malogolowkin et al. [46]	Investigate post-transplant survival	Kaplan–Meier and Cox models	253 relapsed WT patients’ post-chemotherapy and HCT	5-year EFS and OS were 36% and 45%, respectively	Highlighting post-transplant survival challenges informs tailored follow-up protocols and personalized intervention strategies.
Cresswell et al. [47]	Explore genetic heterogeneity within WT	Genome-wide analysis (GWA), MEDICC algorithm	20 WT cases	Multiple samples needed to identify genetic heterogeneity and evolution	Understanding intra-tumor genetic heterogeneity aids in developing personalized risk assessment and treatment modifications.
Cone et al. [48]	Review biomarkers for prognostic significance	PRISMA guidelines, systematic reviews, and meta-analyses	32 biomarkers in 7381 WT patients	11p15 loss of heterozygosity strongly predicts recurrence	Biomarkers help to tailor prognostic evaluations and personalize therapeutic approaches based on individual risk profiles.
Diets et al. [49]	Assess TRIM28 as a prognostic marker	Exome sequencing, tumor DNA analysis	31 WT cases	Identified TRIM28 mutations in germline and somatic samples	TRIM28’s role accentuates the need for personalized molecular diagnostics and tailored therapeutic interventions.
Liu et al. [50]	Analyze the role of miR-144-3p in WT	qRT-PCR, bioinformatics, functional assays	80 WT tissues and matched standard samples	Downregulation of miR-144-3p linked to increased tumor aggressiveness	The miR-144-3p levels are potential personalized markers for monitoring treatment efficacy and adjusting therapeutic strategies.
Fernandez et al. [51]	Assess the association of biomarkers with WT recurrence	Analysis of AREN0532 study cases, biomarker evaluation	116 WT patients	11p15 methylation status correlated with relapse risk	Methylation status could aid in shaping individualized therapeutic strategies by predicting relapse risk.
You et al. [52]	Evaluate lymph node density’s impact on survival	Kaplan–Meier, Cox regression models	1924 WT patients (SEER database)	Lower LND associated with better survival rates	Lymph node density findings inform personalized surgical and therapeutic approaches to boost survival outcomes.
Ortiz et al. [53]	Identify prognostic markers in urine for WT	Mass spectrometry, ELISA	49 WT patients and controls	Urine PHB levels correlated with tumor recurrence and survival	Urine biomarkers provide a non-invasive approach for personalizing prognosis and subsequent treatment pathways.
Taskinen et al. [45]	Examine the histological response to preoperative chemotherapy	Statistical analysis, Wilcoxon signed-rank test	52 WT cases with pre- and post-chemotherapy imaging	Preoperative chemotherapy significantly reduced total and viable tumor volumes	The response to preoperative chemotherapy highlights the need for personalized treatment planning to maximize tumor shrinkage before surgery.
Liu et al. [54]	Identify prognostic biomarkers through data analysis	Differentially Expressed Gene Analysis, Random Survival Forest	RNA-seq and clinical data from the TARGET and GEO databases	Identified gene signatures predictive of WT prognosis	Identified gene signatures offer personalized prognostic information that can guide treatment and monitoring strategies.

## Abbreviations

WT	Wilms tumor
SIOP	The Société Internationale d’Oncologie Pédiatrique (International Society of Pediatric Oncology)
COG	Children’s Oncology Group
NWTS	The National Wilms Tumor Study Group
US	Ultrasonography
CT	Computed Tomography
MRI	Magnetic Resonance Imaging
ddPCR	Digital Droplet Polymerase Chain Reaction
ROC	Receiver Operating Characteristic
SEER database	Surveillance, Epidemiology, and End Results Program
OS	Overall Survival
CSS	Cancer-Specific Survival
AUCs	Area Under the Curve
EFS	Event-Free Survival
HCT	Hematopoietic Cell Transplantation
GWA	Genome-Wide Analysis
PRISMA	Preferred Reporting Items for Systematic Reviews and Meta-Analyses
TAMs	Tumor-Associated Macrophages
ELISA	Enzyme-Linked Immunosorbent Assay
LND	Lymph Node Density
PHB	Prohibitin
GEO	The Gene Expression Omnibus
TARGET	Therapeutically Applicable Research to Generate Effective Treatments
lncRNAs	Long Non-Coding RNAs
IMRT	Intensity-Modulated Radiation
CAR T-Cell treatment	Chimeric Antigen Receptor T-Cell treatment
